# Potential Association between Distal Deep Vein Thrombosis and Asymptomatic Atherosclerosis

**DOI:** 10.1055/s-0041-1741076

**Published:** 2021-12-30

**Authors:** Angelo Adamo, Luca Spiezia, Valle Fabio Dalla, Giampiero Avruscio, Paolo Simioni

**Affiliations:** 1 Department of Medicine, Thrombotic and Hemorrhagic Diseases Unit, Padova University Hospital, Padova, Italy; 2 Department of Cardiac, Angiology Unit, Thoracic and Vascular Sciences, Padova University Hospital, Padova, Italy

**Keywords:** atherosclerosis, deep vein thrombosis, peripheral arterial disease, pulmonary embolism, venous thromboembolism

## Abstract

**Background** Several studies have previously reported an association between idiopathic proximal deep vein thrombosis (DVT) and atherosclerosis, but whether spontaneous distal DVT is associated with asymptomatic atherosclerosis is still unknown.

**Methods** Ultrasonography of the carotid arteries was done for plaque detection and intima-media thickness (IMT) evaluation, and the ankle-brachial index (ABI) in 116 patients with spontaneous DVT and without symptomatic atherosclerosis. Fifty-seven patients (M/F 19/38, age range 54–78 years) had distal DVT and 59 (M/F 24/35, age range 51–73 years) had proximal DVT. A group of 57 (M/F 21/36, age range 64–70 years) matched subjects acted as controls.

**Results** No significant difference was found in carotid plaques between patients with distal or proximal DVT versus controls ( *p* > 0.05 in all comparisons). Carotid IMT (mean ± SD) was significantly increased in patients with distal (1.00 ± 0.20 mm) and proximal (0.98 ± 0.16 mm) DVT versus controls (0.88 ± 0.15 mm, *p* <0.01 in both comparisons). An ABI £ 0.9 was found in 3/57 (5.3%) and 5/59 (8.5%) patients with distal and proximal DVT, respectively versus no controls with abnormal ABI.

**Conclusion** Our results revealed that there may be an association between spontaneous distal DVT and asymptomatic atherosclerosis, and confirmed the known association between idiopathic proximal DVT and asymptomatic atherosclerosis. Larger studies are needed to confirm our results and to evaluate their clinical implications.

Patients with spontaneous venous thromboembolism (VTE) have a higher prevalence of atherosclerosis defined as the presence of asymptomatic atherosclerotic lesions in the carotid arteries as compared with patients with VTE secondary to known risk factors and healthy subjects. 1 In addition, the long-term incidence of cardiovascular disease is reportedly higher in patients with idiopathic VTE. 2 3 These findings suggest that VTE and cardiovascular disorders may share common risk factors and that in some patients at risk for atherosclerosis, VTE might occur as the first symptomatic cardiovascular event. Yet, two large cohort studies have challenged this hypothesis by demonstrating that the presence of atherosclerosis was not predictive of an increased risk of VTE. 4 5 Subsequently, other studies have provided further evidence to support the association between VTE and atherosclerosis. 6 In a case–control study using chest CT scan, Hong et al found a higher prevalence of coronary artery calcium in patients with unprovoked VTE than in matched controls. 7 In a series of almost 24,000 consecutive autopsies, Eliasson et al found an increased prevalence of VTE in patients with arterial thrombosis, except for those with coronary artery thrombosis. 8 Milan et al conducted a case–control study on subjects older than 50 years to assess the prevalence of symptomatic or subclinical atherosclerosis in 100 unselected patients with unprovoked VTE, and compared it with that of 100 patients with provoked VTE and 100 matched controls free from VTE disorders 9 : the prevalence of atherosclerosis was higher in patients with VTE (especially with unprovoked episodes) than in controls. Several studies and a meta-analysis have indicated that subjects with VTE may be at increased risk for acute cardiovascular events, 10 11 12 13 14 15 16 which is consistent with VTE and arterial cardiovascular events sharing common risk factors (e.g., obesity, hypertension, smoking, and diabetes/hyperglycemia). 6 17 18 19

Although the association between venous and arterial thromboembolism has been well described, nearly all the studies published in the literature enrolled only patients with proximal DVT and/or pulmonary embolism. This begs the question of whether those findings also apply to distal DVT, an often underestimated thrombotic disease. Therefore, we conducted a case–control study aiming to evaluate the possible association between distal DVT and asymptomatic atherosclerosis.

## Methods

### Study Design and Objective

We considered all consecutive outpatients evaluated at the Angiology Unit and the Thrombotic and Hemorrhagic Diseases Unit at Padova University Hospital between August 2020 and December 2020 with a recent (within a month) diagnosis of spontaneous proximal or distal DVT, as assessed by compression ultrasonography. The diagnosis of proximal DVT is established by the presence of thrombi in the iliac, femoral and/or popliteal veins. Isolated distal DVT is located below the knee, and confined to the calf veins (peroneal, posterior, anterior tibial, and muscular veins). Exclusion criteria were: recurrent DVT, a history of secondary VTE (e.g., cancer, pregnancy within the previous 3 months; trauma or leg fracture, immobilization for more than 1 week, surgical procedure within the last month or ongoing hormonal treatment), a history of symptomatic atherosclerosis (ischemic stroke, transient ischemic attack, acute myocardial infarction, angina, history of carotid revascularization, or intermittent claudication) and severe liver or kidney failure. All enrolled patients received conventional treatment (i.e., unfractionated or low-molecular-weight heparin followed by oral anticoagulant therapy) according to current guidelines.

We selected 57 subjects without DVT, matched for age and sex with cases, among caregivers and relatives of our cases who acted as controls. All enrolled patients provided written informed consent in compliance with the principles of the Declaration of Helsinki and the Padova University Hospital's Ethical Committee was notified as required for observational studies.

Before undergoing carotid ultrasonography or ankle-brachial index (ABI) measurement, our entire study population was evaluated for the presence of risk factors for atherosclerosis. In particular, data on the following variables were recorded on a standard form: smoking status, with smoking defined as habitual daily use of 10 cigarettes, with interruptions of less than 1 month; hypertension, defined as a finding of office systolic blood pressure (SBP) values 140 mm Hg and/or diastolic blood pressure values 90 mm Hg on at least two occasions, 20 or by the use of hypotensive drugs; obesity, defined as a body-mass index (the weight in kilograms divided by the square of the height in meters) 30; diabetes mellitus, defined as an 8-hour fasting plasma glucose level 126 mg/dL (7.0 mmol/L) or a level 200 mg/dL (11.1 mmol/L) 2 hours after the administration of 75 g of oral glucose or randomly in patients with classic symptoms of hyperglycemia or hyperglycemic crisis or a level of glycated hemoglobin A1c 6.5% (48 mmol/mol) 21 or by the use of antidiabetic drugs; hyperlipidemia defined as a fasting venous cholesterol level >240 mg/dL (6.19 mmol/L), or a fasting venous triglyceride level >150 mg/dL (1.69 mmol/L), according to our laboratory upper limit, on at least two occasions, or by the use of lipid-lowering drugs.

### Carotid Ultrasonography

Bilateral assessment of the carotid arteries was performed by a trained sonographer. The test was performed using an EPIQ 5 device (Philips) with an automatic intima-media thickness (IMT) measuring module and a 2 to 22 MHz linear probe for B-mode imaging and pulsed-wave color Doppler imaging according to standardized methods. 22 Patients were examined in supine position with a 45-degree neck rotation in the opposite direction of the site being examined. The carotid trunk was identified using both B-mode and pulsed-wave color Doppler ultrasonography, and the following segments were examined: common carotid artery, carotid bifurcation, and internal and external carotid arteries. All arteries underwent longitudinal and transverse scans as well as flow analysis.

An ultrasound of both walls of the common carotid artery in a longitudinal image reveals two parallel echogenic lines which correspond to the lumen-intima and media-adventitia interfaces. The IMT is measured as the distance between the two lines in compliance with the IMT correlation studies comparing this ultrasound pattern to carotid anatomy specimens. 23 Plaques are focal structures encroaching upon the arterial lumen by at least 0.5 mm or 50% of the surrounding IMT value, or demonstrates IMT >1.5 mm.

IMT values of the right and left common carotid arteries were measured on the far wall of the common carotid artery at least 5 mm below its end along approximately 10 mm length of a straight arterial segment and expressed in millimeters. 24 The higher of the two values was recorded. IMT >0.9 mm was categorized as abnormal according to the latest ESC/ESH Hypertension Guidelines. 20 24

The percentage of vessel obstruction was measured along the longitudinal axis and classified as follows: normal, <50%, 50 to 69%, 70%, near occlusion, total occlusion according to the European Carotid Surgery Trial criteria and Doppler US Criteria for Diagnosis of Internal Carotid Artery Stenosis. 25 When more than one plaque was found, the greatest degree of obstruction along with the number and site of associated ipsilateral and contralateral smaller lesions was recorded.

### Ankle-Brachial Index

The ABI is measured in supine position by a trained technician, using a pressure cuff in both arms and near each ankle. The SBP is measured after a 5 to 10 minute rest using a Doppler ultrasound probe (2–22 MHz) at the posterior and anterior tibial (or dorsal pedis) arteries of each foot and at the brachial artery of each arm. The ABI is calculated by dividing the highest pressure recorded at the ankle by the highest pressure recorded at the brachial artery; an ABI £ 0.9 is considered diagnostic for occlusive peripheral arterial disease. 26

### Statistical Analysis

The sample size calculation was based on previous observations and the following assumptions: (1) expected Δ IMT = 0.02 mm between thrombotic patients and controls; (2) expected SD = 0.5 mm; ( 3 ) power = 90%; (4) *α* = 0.05. Therefore, we needed two groups (e.g., thrombotic patients and controls) of at least 55 patients each. Categorical variables were described as frequencies, and comparisons were performed by Fisher's exact test. The normality assumption was assessed by Shapiro–Wilk normality test. The ANOVA test was performed for parametric variables and the Kruskal–Wallis test was used for non-parametric variables. Items of interest were assessed by logistic regression analyses. Results were expressed as odds ratios (OR) with 95% confidence intervals (CIs). All statistical analyses were performed using GraphPad Prism 7 (GraphPad Software Inc., California, United States) and the PAWS Statistics 17.0.2 (SPSS Inc.) for Windows.

## Results

Among 207 patients consecutively evaluated during the study period, 116 were enrolled: *n* = 57 patients (M/F 19/38, age range 54–78 years) had distal DVT and *n* = 59 (M/F 24/35, age range 51–73 years) had proximal DVT. During the same time period, *n* = 57 (M/F 21/36, age range 64–70 years) matched subjects without DVT were enrolled as controls. Table 1 shows the main characteristics of the study subjects. No significant difference was observed among the three groups regarding the prevalence of risk factors.

**Table 1:** Main characteristics of the study population

	Distal DVT ( *N* = 57)	Proximal DVT ( *N* = 59)	Controls ( *N* = 57)	Distal DVT vs. controls	Proximal DVT vs. controls
Age − year ± SD	66.6 ± 12.25	62.1 ± 11.63	62.2 ± 8.48		
Men sex no. (%)	19 (33.3)	23 (40.7)	21 (36.8)	p 0.6947	p 0.6717
Body mass index ± SD	26.78 ± 3.98	28.26 ± 5.68	26.81 ± 3.96		p 0.1436
Obesity (BMI ≥30), no. (%)	12 (21.1)	20 (33.9)	15 (26.3)	p 0.5087	p 0.3738
Smoker, no. (%)	13 (22.8)	8 (13.6)	6 (10.5)	p 0.0785	p 0.6162
Ex-smoker, no. (%)	12 (21)	20 (33.9)	11 (19.3)	p 0.8155	p 0.0757
Diabetes, no. (%)	7 (12.3)	5 (8.5)	6 (10.5)	p 0.7683	p 0.7061
On treatment, no. (%)	5 (71.4)	4 (80)	5 (83.3)	p 0.6115	p 0.8865
Hypertension, no. (%)	34 (59.6)	26 (44.1)	25 (43.9)	p 0.0916	p 0.9820
On treatment, no. (%)	32 (94.1)	25 (96.1)	24 (96.0)	p 0.7450	p 0.9774
Hyperlipidemia, no. (%)	21 (36.8)	15 (25.4)	13 (22.8)	p 0.1015	p 0.6634
On treatment (statins), no. (%)	14 (66.7)	10 (66.7)	8 (61.5)	p 0.7611	p 0.7776
On antiplatelets	6 (10.5)	0 (0)	4 (7.0)	p 0.5079	

### Carotid Plaques

At least one carotid plaque was detected in 38/57 (66.7%) patients with distal DVT, 33/59 (55.9%) patients with proximal DVT, and 29/57 (50.9%) controls. In the univariate logistic regression analysis, patients with distal DVT were more likely to have carotid plaques versus controls (OR 1.93; 95% CI, 0.91–4.12; *p* = 0.087); patients with proximal DVT had a slightly higher risk of atherosclerotic lesions versus controls (OR 1.23; 95% CI, 0.59–2.54; *p* = 0.585), though the differences were not statistically significant in either comparison.

After adjusting for the presence of bilateral/unilateral lesions or the greatest degree of obstruction, the odds in patients with distal DVT as compared with those with proximal spontaneous thrombosis and with controls did not differ significantly ( Table 2 ).

**Table 2:** Number of patients with at least one atherosclerotic plaque, carotid involvement (unilateral/bilateral), and grade of maximum stenosis

	Distal DVT ( *N* = 57)	Proximal DVT ( *N* = 59)	Controls ( *N* = 57)	Distal DVT vs. controls OR (95% CI)	Proximal DVT vs. controls OR (95% CI)
Carotid plaques					
Presence, no. (%)	38 (66.7)	33 (55.9)	29 (50.9)	1.93 (0.91–4.12) p 0.087	1.23 (0.59–2.54) p 0.585
Bilateral, no. (%)	23 (60.5)	17 (51.5)	13 (44.8)	1.89 (0.71–5.02) p 0.202	1.31 (0.48–3.56 p 0.599
Stenosis					
Normal, no. (%)	19 (33.3)	26 (44.1)	28 (49.1)	1	1
< 50%, no. (%)	38 (66.7)	32 (54.2)	28 (49.1)	2.00 (0.94–4.28) p 0.072	1.23 (0.59–2.57) p 0.580
50–69%, no. (%)	0 (0)	1 (1.7)	1 (1.8)	–	1.08 (0.06–18.12) p 0.959
≥ 70%, no. (%)	0 (0)	0 (0)	0 (0)	–	–
Near occlusion, no. (%)	0 (0)	0 (0)	0 (0)	–	–
Total occlusion, no. (%)	0 (0)	0 (0)	0 (0)	–	–
Intima-media thickness					
> 0.9 mm, no. (%)	38 (66.7)	42 (71.2)	27 (47.4)	2.22 (1.04–4.74) p 0.037	2.75 (1.28–5.91) p 0.009
Bilateral, no. (%)	27 (71.0)	28 (66.7)	15 (55.6)	1.96 (0.70–5.52) p 0.198	1.60 (0.59–4.32) p 0.353

### Intima-media Thickness

**Fig. 1:**
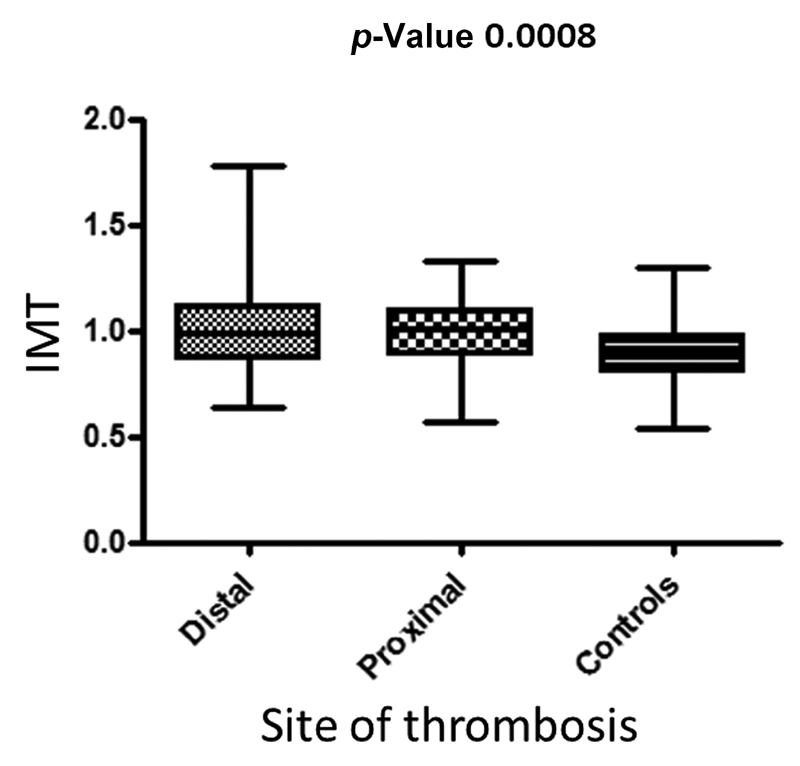
Mean IMT among the three study groups.

The IMT (mean ± SD) was significantly higher in patients with distal (1.00 ± 0.20 mm) and proximal (0.98 ± 0.16 mm) DVT versus controls (0.88 ± 0.15 mm, *p* < 0.01 in both comparisons) ( Fig. 1 ). Interestingly, patients with distal DVT showed a significantly abnormal mean IMT (> 0.9 mm) versus controls (OR 2.22; 95% CI, 1.04–4.74; *p* = 0.0374); we found similar results in the comparison between proximal DVT and controls (OR 2.75; 95% CI, 1.28–5.91; *p* = 0.009) ( Table 2 ).

### Ankle-Brachial Index

**Fig. 2:**
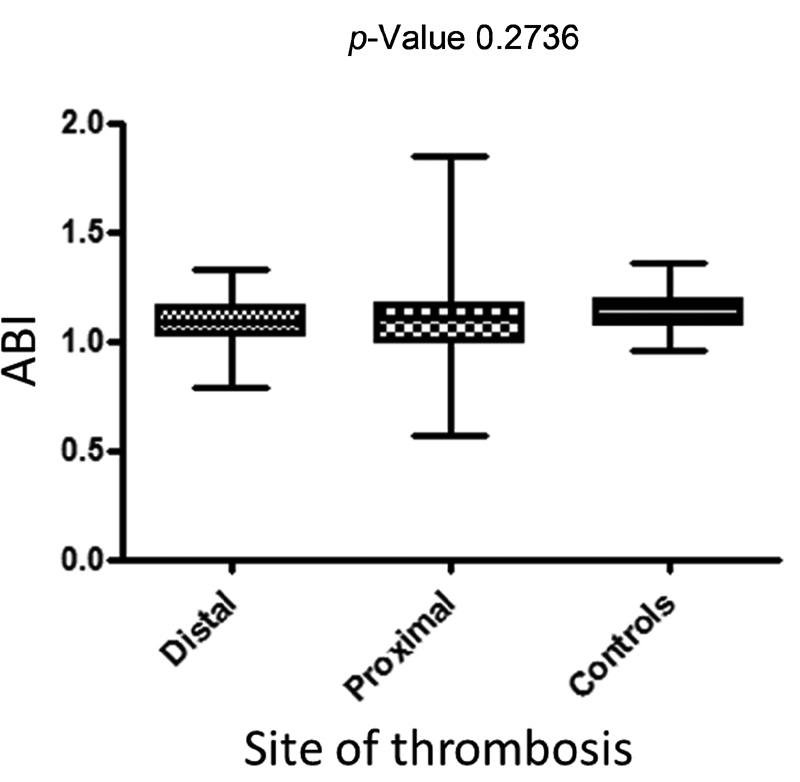
Mean lowest ankle-brachial index (ABI) among the three study groups.

The mean ABI was 1.09 ± 0.11 in patients with distal DVT, 1.09 ± 0.16 in those with proximal DVT, and 1.12 ± 0.09 in controls. No significant difference was found among the three groups ( Fig. 2 ). An ABI £0.9 was detected in 3/57 (5.3%) patients with distal DVT, 5/59 (8.5%) patients with proximal DVT, and no controls ( Table 2 ).

### Discussion

Our findings revealed a possible association between both distal and proximal DVT, and asymptomatic atherosclerotic lesions. The prevalence of abnormal IMT (>0.9 mm) was significantly higher in patients with unexplained distal or proximal thrombotic events (66.7 and 71.2%, respectively) versus age- and sex-matched subjects without thrombosis (47.4%). An analysis of the absolute mean IMT among the three groups indicated an even stronger association. In addition, other features of atherosclerosis (such as the presence of atherosclerotic plaques in the carotid arteries and the extent of contralateral carotid involvement) and signs of occlusive peripheral arterial disease (i.e., ABI £ 0.9) were more frequent among subjects with spontaneous thrombosis than in controls, albeit with no significant differences with respect to the site of thrombosis (distal or proximal).

Our findings on proximal DVT are in line with previous case–control studies and a meta-analysis, 10 11 12 13 14 15 16 as well as a 2003 pilot study by Prandoni et al. 1 It bears noting that an ABI £ 0.9 may also be an indicator of atherosclerosis at other vascular sites, and constitute a prognostic marker for cardiovascular events and functional impairment, even without any symptoms of peripheral arterial disease. 27 28 29 In fact, Libertiny and Hands reported that ABI is linked to an increased VTE diagnosis. 30 Nearly all the aforementioned studies enrolled only patients with either proximal venous thrombosis or pulmonary embolism. By opting to enroll patients with a recent diagnosis of spontaneous isolated distal DVT in our study, we were able not only to confirm the previously well-described venous-arterial link but also to put under a new light this subtype of venous thrombotic localization, often underestimated.

We would be remiss not to mention some of the limitations of our study, and namely, its retrospective design and our relatively small sample size which did not allow us to perform a multivariate analysis. Nevertheless, we found overlapping data as it pertains to arterial involvement between patients with distal and proximal venous thrombosis. Moreover, we found a significant independent relationship between distal venous thrombosis and asymptomatic atherosclerotic disease. From a clinical standpoint, physicians may envision a diagnostic approach that encompasses carotid ultrasound and ABI measurement to early identify asymptomatic peripheral arterial disease in patients with distal and proximal DVT, and thus mitigate the risk of potentially dangerous complications. Furthermore, patients with VTE could be systematically assessed for asymptomatic atherosclerosis to identify common atherosclerotic risk factors such as hypertension, dyslipidemia, smoking habits, and diabetes and aggressively mitigate the risk profile in those with abnormal test results.

Since it is well known that venous and arterial thrombosis share common risk factors, larger studies are needed to ascertain whether to systematically extend the study of arterial diseases to all patients with VTE.

## Notes

**What Is Known on This Topic**

- There is an association between spontaneous venous thrombosis and atherosclerotic disease.
- The indication for anticoagulation in patients with isolated distal thrombosis is debated.

**What Does This Paper Add?**

- Our study shows this association even in case of distal isolated thrombosis.
- Regardless of the chosen treatment it could be reasonable to assess their cardiovascular risk factors.
